# The Role of Nitric Oxide in Cancer Treatment: Ally or Foe?

**DOI:** 10.3390/molecules30132802

**Published:** 2025-06-29

**Authors:** Angelika Myśliwiec, Dorota Bartusik-Aebisher, David Aebisher

**Affiliations:** 1 Department of Biochemistry and General Chemistry, Medical Faculty, Collegium Medicum, University of Rzeszów, 35-959 Rzeszów, Poland; amysliwiec@ur.edu.pl (A.M.); dbartusikaebisher@ur.edu.pl (D.B.-A.); 2 Department of Photomedicine and Physical Chemistry, Medical Faculty, Collegium Medicum, University of Rzeszów, 35-959 Rzeszów, Poland

**Keywords:** nitric oxide, cancer therapy, PDT, iNOS, NOS

## Abstract

Nitric oxide (NO), the first gaseous molecule identified as a signaling mediator, plays a pivotal role in numerous physiological processes including cardiovascular regulation, immune response, and neurotransmission. Synthesized from L-arginine by nitric oxide synthase (NOS), NO exerts both protective and cytotoxic effects depending on its local concentration. At low levels, NO supports tumor growth by mitigating oxidative stress, while at high concentrations, it induces apoptosis through mechanisms such as p53 activation, cytochrome c release, and peroxynitrite formation. These dual properties position NO as a complex but promising agent in cancer therapy. Recent studies have highlighted the potential of NO in enhancing the efficacy of photodynamic therapy (PDT), where it synergizes with reactive oxygen species (ROS) to induce cytotoxic effects in tumor cells. Despite its promise, challenges such as rapid diffusion and limited tumor accumulation hinder NO’s therapeutic utility. This has spurred the development of NO donors and nanotechnology-based delivery systems to enable controlled, site-specific release. Moreover, NO has been shown to counteract multidrug resistance, improve tumor perfusion by dilating vasculature, and potentiate ROS-based therapies like PDT and radiotherapy. However, an emerging concern is NO’s role in promoting proliferation and migration of non-targeted “bystander” tumor cells following PDT-induced stress, primarily through iNOS upregulation. This feedback loop can contribute to tumor aggressiveness and metastasis, underscoring the need for a deeper understanding of NO’s molecular actions. While iNOS inhibitors show preclinical promise in various inflammatory and neoplastic conditions, no such agents have reached clinical approval, due to the complexity and context-dependent effects of NO. Future research should focus on refining NO delivery systems, developing selective iNOS inhibitors, and elucidating NO’s dual role in cancer biology to fully harness its therapeutic potential in PDT and beyond.

## 1. Introduction

Nitric oxide (NO) is a unique signaling molecule in the human body, belonging to the gases. It is formed from the amino acid L-arginine through an enzymatic reaction catalyzed by nitric oxide isoforms (NOSs). The discovery of NO as a biologically active substance was groundbreaking and surprising to the scientific community, since most previously known physiological regulators—such as hormones or neurotransmitters—were protein, lipid, or belonged to other groups of chemical compounds, but none of them were gases. Nitric oxide, as a simple inorganic compound, has significantly changed our understanding of cell signaling processes [1,2].

NO interacts with many enzyme systems in the body and has the ability to bind to heme, an iron-porphyrin cofactor found in important enzymes such as guanylyl cyclases and oxidases. Thanks to these properties, NO influences the activity of numerous signaling pathways and acts similarly to hormones, regulating a number of important physiological functions [3,4]. NO acts as a versatile cellular mediator whose mechanisms of action include both classical signaling pathways and less conventional forms of molecular modification. As a signaling molecule, NO exhibits the ability to interact with numerous cellular components, making it an important regulator of physiological homeostasis and pathological processes such as inflammation, tumorigenesis, and oxidative stress [5,6]. One of the best understood and fundamental mechanisms of NO action is its interaction with soluble guanylyl cyclase (sGC). NO binds to the heme prosthetic group of this enzyme, containing an iron ion in the Fe^2+^ state, leading to its activation and catalyzing the cyclization of guanosine triphosphate (GTP) to cyclic guanosine monophosphate (cGMP). The resulting cGMP acts as a second messenger, activating protein kinase G (PKG), which in turn regulates intracellular calcium levels. A decrease in Ca^2+^ ion concentration results in a number of cellular responses, including vascular smooth muscle relaxation, inhibition of platelet aggregation, increased vascular permeability, and modulation of vascular tone. Because of this signaling pathway, NO is considered a key factor in the regulation of blood pressure and endothelial function. It is noteworthy that among the various forms of guanylyl cyclase, only sGC is a direct molecular target for NO, which distinguishes it as the main transmitter of its signal in cells. However, NO is not limited to this pathway alone. Under conditions of oxidative stress, NO can react with superoxide anion (O_2_^−^) to form the highly reactive peroxynitrite (ONOO^−^), which is capable of damaging lipids, proteins, and nucleic acids. The products of this reaction, such as 3-nitrotyrosine, can be persistent markers of nitrosative stress and cellular dysfunction [7,8]. In addition to the sGC-cGMP mechanism, NO affects protein function through direct modifications of amino acid residues. One of the most common is S-nitrosylation—a reversible modification of cysteine residues involving the attachment of an NO group to a thiol group, which can affect enzymatic activity, stability, and protein localization [9]. High levels of oxidative stress can lead to irreversible tyrosine nitration, resulting in, among other things, conformational changes or inhibition of signaling protein activity, as documented in the context of the MAPK pathway and Src kinase [10,11,12,13].

In addition, NO can modulate the activity of key signaling enzymes, such as cyclooxygenases (COX), protein kinases, including protein kinase C (PKC), or apoptotic enzymes, thereby affecting inflammation, proliferation, and cell death. By regulating the translation, translocation, and stability of signaling proteins, NO also plays an important role in intercellular communication and adaptive cellular responses [14]. One of the best-known effects of NO is its role in vasodilation. It works by relaxing vascular smooth muscle, leading to lower blood pressure and improved blood flow. This process happens very quickly—nitric oxide acts almost immediately upon release, but is just as quickly neutralized and removed, making its presence in the blood last only a few seconds. This short duration of action makes NO ideal for precise and momentary regulation of vascular function [2,8,15].

NO synthesis occurs mainly in endothelial cells, where the enzyme endothelial-type nitric oxide synthase catalyzes the conversion of the amino acid L-arginine to L-citrulline and NO, in the presence of cofactors such as NADPH, BH_4_, FAD, FMN, and oxygen. The activity of eNOS is regulated by various mechanical and chemical stimuli—including shear stress, the presence of acetylcholine, bradykinin, insulin, or vascular endothelial growth factor (VEGF). NO, as a small and lipophilic gaseous molecule, diffuses rapidly across cell membranes into neighboring smooth muscle cells. Inside muscle cells, NO binds to the heme group of soluble guanylate cyclase, activating this enzyme and causing the conversion of GTP to cGMP. The increase in cGMP levels activates protein kinase G (PKG), which initiates a series of biochemical reactions leading to a decrease in the concentration of calcium ions in the cytoplasm (including through inhibition of calcium channels, activation of calcium pumps, and opening of potassium channels). The effect is inactivation of myosin light chain kinase and inhibition of actin–myosin interaction, resulting in vascular smooth muscle relaxation. As a result, blood vessels dilate, resulting in lower blood pressure and improved tissue perfusion—especially in organs with high oxygen demand, such as the brain, heart, and kidneys. The mechanism is also important in a pharmacological context—for example, nitroglycerin and other NO donors are used in the treatment of angina pectoris because they cause rapid coronary vasodilation and reduce cardiac workload [16,17,18].

Endothelial NO deficiency—caused, for example, by eNOS dysfunction, oxidative stress, or inflammation—is closely linked to the pathogenesis of cardiovascular diseases such as atherosclerosis, hypertension, type 2 diabetes, or chronic renal failure. In contrast, excessive production of NO, particularly by the inducible form of iNOS under conditions of sepsis or chronic inflammation, can lead to severe hypotension and impaired organ perfusion. For this reason, nitric oxide is considered an ambiguous biological signaler—its adequate levels are a prerequisite for vascular health, while both deficiency and excess can lead to serious clinical consequences [19,20,21].

In addition, NO plays an important role in preventing blood clots—it inhibits platelet aggregation (clumping), which reduces the risk of intravascular clots. It also has an anti-inflammatory effect, reducing the adhesion of leukocytes (white blood cells) to blood vessel walls, which is important in controlling inflammatory processes and protecting the endothelium. Abnormalities in the production or function of NO are often observed in the course of various chronic diseases, such as diabetes, arteriosclerosis, and hypertension. In these conditions, nitric oxide-related signaling pathways are impaired, resulting in impaired blood vessel function, increased risk of blood clots, and increased inflammation [22].

For these reasons, NO has become the subject of intense clinical and pharmacological research. It is being considered as a therapeutic target for the treatment of many cardiovascular diseases, as well as in neurology, oncology, and immunology. Some drugs—such as nitroglycerin used in angina—work precisely by releasing NO or stimulating its signaling pathway. Also, PDE5 inhibitors, such as sildenafil, indirectly increase NO levels by promoting vasodilation in specific tissues [23].

**Figure 1:**
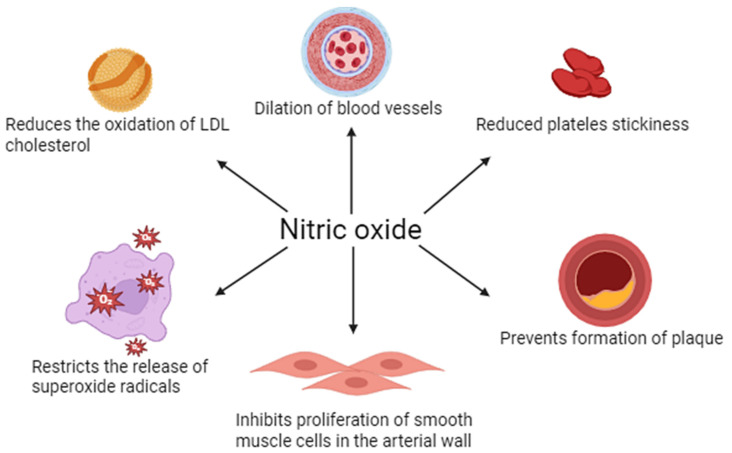
Physiological importance of nitric oxide in vascular endothelial function.

NO plays a key role in regulating vascular endothelial function. It is mainly synthesized by eNOS in response to mechanical (e.g., blood shear) and chemical (e.g., acetylcholine) stimuli. NO penetrates into vascular smooth muscle, where it activates guanylate cyclase, increasing the concentration of cGMP and leading to vasodilation (vasodilation). In addition, NO inhibits leukocyte adhesion and activation, platelet aggregation, and smooth muscle proliferation, making it an important protective factor in maintaining vascular homeostasis and counteracting the development of atherosclerosis (Figure 1).

In living organisms, it is produced by enzymes of the NOS group, which include three main isoforms: neuronal nitric oxide isoform (nNOS or NOS1), inducible nitric oxide isoform (iNOS or NOS2), and endothelial nitric oxide isoform (eNOS or NOS3). These enzymes are involved in a biochemical reaction in which L-arginine, NADPH (nicotinamide dinucleotide phosphate), and oxygen (O_2_) are converted into the end products L-citrulline and nitric oxide [24,25]. Each of these isoforms has specific biological functions. For example, NO synthesized by nNOS plays a key role in processes related to the transmission of nerve signals—particularly in the mechanisms of so-called retrograde neurotransmission, or feedback communication between nerve cells. In turn, NO produced by eNOS is essential for the proper regulation of blood pressure, as it acts as a powerful vasodilator, promoting blood flow [24].

**Figure 2:**
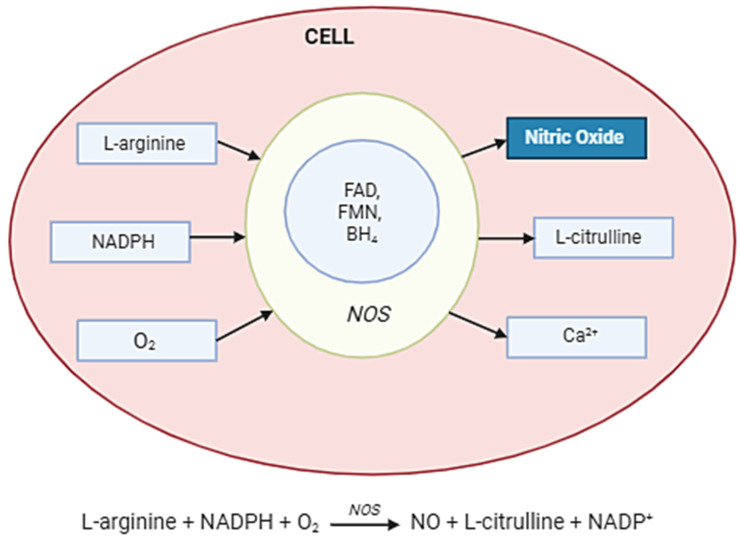
Nitric oxide synthesis in the cell. Abbreviations: NOS enzyme—nitric oxide synthase; Arginine (L-arginine)—the main substrate; O_2_—molecular oxygen; NADPH—as an electron donor; BH_4_—tetrahydrobiopterin; FAD, FMN, hem—cofactors and auxiliaries; NO—diffuses across the membrane; Citrulline—a by-product.

NO is synthesized in cells by NOSs, which convert the amino acid L-arginine into NO and L-citrulline in the presence of oxygen and cofactors such as NADPH, tetrahydrobiopterin (BH_4_), and flavins (FAD, FMN). The three NOS isoforms—neuronal (nNOS), endothelial (eNOS), and inducible (iNOS)—differ in regulation and expression. While eNOS and nNOS are calcium/calmodulin-dependent and produce small amounts of NO for signaling, iNOS is induced under inflammatory conditions and generates high, sustained levels of NO. Once produced, NO diffuses rapidly across membranes to exert its effects, including activation of soluble guanylate cyclase (sGC) and modulation of cellular signaling pathways (Figure 2).

In the context of oncology, however, nitric oxide has a more complex and often adverse function. Studies have shown that in cancerous conditions, when its concentration is maintained at low to moderate levels (in the range of 0.1–0.5 micromoles), NO can contribute to the resistance of cancer cells to the anticancer therapies used. In addition, it acts as a signaling molecule that promotes proliferation (i.e., proliferation) and migration of cancer cells, which can increase tumor aggressiveness [26,27]. Inducible iNOS, which shows elevated levels of expression in many types of cancer cells, plays an important role in these processes. What is more, these levels can be further increased in response to drugs that induce cellular stress, such as cisplatin—a common cytostat used in cancer therapy [28].

Nitric oxide produced by iNOS plays an important role in the elimination of microorganisms and the destruction of cancer cells. Its mechanism of action is based on the formation of reactive nitrogen species (RNS), such as peroxynitrite (ONOO^−^), which is formed from the reaction of NO with superoxide anion radical (O_2_^−^). These highly reactive molecules lead to damage of proteins, lipids, and nucleic acids of microorganisms, which contributes to their elimination. At the same time, iNOS indirectly shapes the immune response by modulating the activity of other immune cells, such as by inhibiting T cell proliferation or promoting apoptosis of target cells [29,30].

However, excessive or chronic iNOS activity can lead to pathological consequences. In conditions of chronic inflammation, as seen in inflammatory bowel disease (IBD), asthma, hepatitis, or autoimmune diseases (e.g., systemic lupus erythematosus), overproduction of NO and peroxynitrite can damage host tissues, contributing to worsening inflammation and disease progression. Studies in animal models have shown that blockade of iNOS—either genetically or pharmacologically (e.g., with selective inhibitors such as L-NIL)—leads to reduced inflammation and improved tissue integrity [31,32].

Therefore, iNOS remains not only an important component of physiological immune defense, but also a potential therapeutic target. Inhibitors of iNOS are currently under investigation in the context of the treatment of chronic inflammation, sepsis, as well as in neuroinflammatory diseases such as multiple sclerosis and Alzheimer’s disease, where chronic activation of microglia leads to NO-mediated neurotoxicity [33].

In the context of physiological cell signaling, NO belongs to a group of small bioactive molecules that exhibit potent effects at very low concentrations. Typical physiological concentrations of NO are in the range of 100–500 nM and are considered low, although even at these values the molecule plays a key role in the regulation of vascular tone, neurotransmission, and the immune response. In comparison, another reactive signaling molecule—H_2_O_2_ cells—operate in a wide range of 1 to 1000 nM, which depends on the cell type and oxidative stress conditions. In contrast, cGMP, which is a secondary messenger activated by NO through guanylate cyclase stimulation, is typically maintained at 1–100 nM. This comparison shows that NO acts in similar or lower concentration ranges than other small-molecule mediators, but its biological efficacy is due to its high reactivity and short half-life, allowing for precise and local regulation of cellular functions [34,35]. Table 1 shows the biological significance of nitric oxide.

## 2. Materials

### 2.1. Literature Search Strategy

To identify relevant studies on the prognostic role of inducible iNOS in malignancies, a comprehensive and systematic search of three major databases was conducted: PubMed National Library of Medicine, Bethesda, MD, USA, Web of Science, and Embase. The search included all available publications from 2015 to 5 June 2025. Logical combinations of English search terms were used, including both different names of the target enzyme and terms related to cancer and patient prognosis. The keywords used were as follows: “inducible nitric oxide synthase”, “iNOS”, ‘NOS2′ (to denote the enzyme), “cancer”, “tumor”, “carcinoma”, “neoplasm”, and “prognosis”, “survival”, “outcome” (to denote endpoints). Terms were combined using logical operators “AND” and “OR” to cover the widest possible range of literature. In addition, a manual analysis of the bibliographies of all papers eligible for full-text review was performed to search for additional articles that might meet the eligibility criteria but were not caught in the initial electronic search.

### 2.2. Eligibility and Exclusion Criteria

For a publication to be eligible for the final meta-analysis, it had to meet all of the following inclusion criteria: the subject of the study had to address the association between iNOS (NOS2) expression and the prognosis of patients with malignant tumors, regardless of tumor location; the article had to include quantitative data in the form of hazard ratios (HRs) along with 95% confidence intervals (CIs) derived from multivariate analysis, ensuring control for confounding variables; the study population had to be clearly divided into groups according to the level of iNOS expression: high/positive vs. low/negative. In turn, publications were excluded from the analysis that were in the nature of letters to the editor, editorial comments or case reports; did not involve human studies (e.g., experiments on animal models, cell lines); did not provide numerical data to calculate HRs; or did not present results in a way that allowed them to be used in a meta-analysis.

### 2.3. Data Extraction Process and Quality Assessment

Data from eligible articles were independently reviewed and analyzed by two investigators according to a pre-designed protocol and standardized extraction form. In cases of divergent assessments, the researchers engaged in a joint discussion to reach a consensus. If discrepancies persisted, a final decision was made with the approval of a third reviewer. The following information was extracted from each included study: the author and year of publication; the country and geographic region where the study was conducted; the duration of the study and the number of participants; the type of cancer involved; the mean age of patients, possibly by group; the method used to determine iNOS expression levels (e.g., immunohistochemistry, RT-PCR); the cut-off value used to classify the expression level; the length of the follow-up period; the risk ratios (HRs) and corresponding 95% CIs for the main endpoints, such as overall survival (OS), cancer-specific survival (CSS), relapse-free survival (RFS). Only data from multivariate (multivariate) analyses were included to maximize the reliability and comparability of results in the meta-analysis.

## 3. Results

### 3.1. Nitric Oxide in Different Types of Cancer

Under sustained, high concentrations of NO, typically at micromolar levels produced by inducible iNOS in activated inflammatory macrophages, NO can exert cell-damaging effects. Its reactivity means that at such concentrations, the compound can damage DNA, proteins, and other cellular structures, leading to genetic mutations that promote the development of cancer (mutagenic and carcinogenic effects). On the other hand, high concentrations of NO can also have a cytotoxic effect—destructive against mature, already formed cancer cells, which may be part of the body’s immune response [39]. The opposite occurs at lower NO concentrations, in the nanomolar to low micromolar range. Such levels, also generated by iNOS, but in a different context of the tumor environment (e.g., in tumor cells or cells of the tumor microenvironment), may have tumor-promoting functions. In this regard, NO acts as a signaling mediator, activating a number of molecular pathways involved in cell proliferation, migration, angiogenesis, and inhibition of apoptotic mechanisms. The effect is to increase the survival of cancer cells, their ability to invade, and their metastatic potential [39,40,41]. Table 2 shows the role of NO in various types of cancer, highlighting its diverse effects on tumor progression, angiogenesis, metastasis, and response to therapy. The table summarizes current research findings on how NO can act both as a tumor promoter and as an inhibitor, depending on factors such as concentration, cellular context, and tumor microenvironment. Additionally, it outlines specific molecular pathways influenced by NO and its impact on different cancer types, providing insight into potential therapeutic targets involving NO modulation.

Activation of these pathways often occurs through so-called S-nitrosation, which is the attachment of the NO group to the thiol group (-SH) of cysteine residues in proteins. This process changes the function of the protein—most often by increasing its activity or protecting it from degradation. S-nitrosation is usually the result of transnitrosation, i.e., the transfer of a nitroso group from other compounds such as S-nitrosoglutathione (GSNO), rather than the direct action of NO [50]. This protein modification affects many cellular effectors, including JNK and ASK1 kinases, which are normally involved in the activation of apoptotic pathways, but are inhibited upon S-nitrosation, promoting the survival of cancer cells; Bcl-2 protein, which has an anti-apoptotic function; its stabilization by NO inhibits its degradation and further promotes cell resistance to cell death; PTEN, a tumor suppressor protein that inhibits the PI3K/Akt pathway; S-nitrosation leads to its inactivation, eliminating the anti-tumor mechanism [51,52,53].

**Figure 3:**
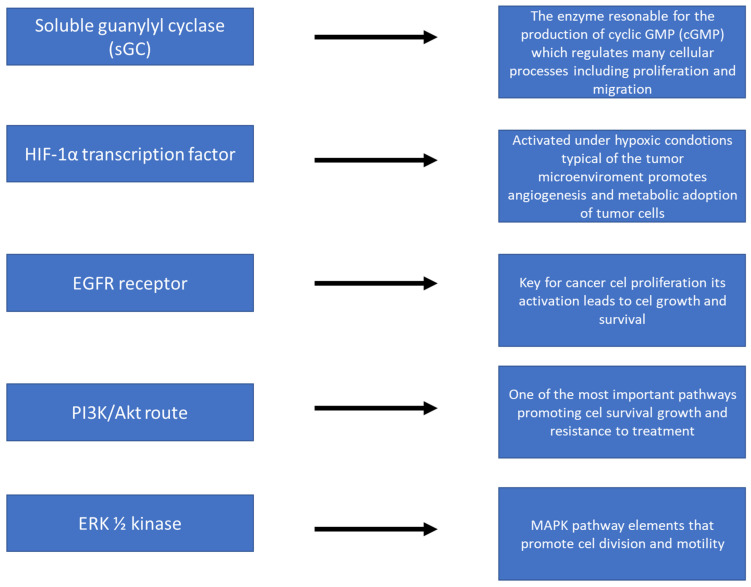
NO action at the molecular level—activation of signaling pathways.

Figure 3 shows the action of nitric oxide (NO) at the molecular level, focusing on the activation of key signaling pathways in cancer cells. NO, as a small signaling molecule, can diffuse across cell membranes and affect a variety of biological processes. The illustration shows how NO activates signaling cascades such as the PI3K/Akt pathway, MAPK/ERK, or NF-κB, which are responsible for, among others, cell proliferation, survival, angiogenesis, and resistance to oxidative stress. Also shown are NO interactions with molecules such as guanylate cyclase (sGC) and an increase in cGMP levels, which influence further intracellular reactions. In addition, the figure shows the effect of NO on the expression of genes related to inflammation, cancer progression, and immune response. The whole illustrates the complex, often context-dependent nature of NO in the regulation of cancer processes.

In many tumors with *TP53* gene mutations, increased iNOS expression is observed, leading to elevated nitric oxide (NO) levels, which is associated with a more aggressive tumor phenotype—increased cell proliferation, migration, and invasiveness. Loss of p53 function promotes the exploitation of NO’s pro-cancer effects. The expression level of iNOS is now recognized as an important prognostic biomarker—its elevated values correlate with a worse disease course and lower patient survival. iNOS is usually absent in healthy tissues, but is strongly induced under the influence of inflammatory cytokines (e.g., TNF-α, IL-1β, IFN-γ), oxidative stress, and pathogens. Studies on tissues from patients with IBD and colorectal cancer have shown high expression of iNOS and correlating levels of nitrotyrosine, a marker of nitrosative stress. In the HT-29 cell model, inflammatory stimulation and the presence of peroxynitrite were confirmed to increase iNOS expression and NO production. Chronic low concentrations of NO—in contrast to toxic doses—promote tumor survival, proliferation, migration, and resistance to treatment, including through activation of PI3K/Akt, MAPK/ERK, HIF-1α pathways and inhibition of apoptosis. Induction of iNOS may also be an adaptive response to therapeutic stress (chemotherapy, radiation therapy), as confirmed in breast, prostate, and glioblastoma cancer models, where NO promoted the development of a treatment-resistant phenotype. NO also affects the immune environment of the tumor: it promotes the secretion of cytokines (TNF-α, IL-6, IL-8), reprograms macrophages to an M2 phenotype, and promotes T-reg expansion, leading to tumor immunotolerance. Experimental models in mice (p53−/− and NOS2+/+) showed that *C. parvum*-induced inflammation accelerated tumor growth, pointing to NO as an important promoter of carcinogenesis [50,54,55,56,57,58,59,60,61,62].

NO plays a complex and bidirectional role in glioma pathogenesis, depending on its concentration, exposure time, and the local tumor microenvironment. At low, chronic concentrations, NO exerts a pro-tumor effect by promoting proliferation, migration, and resistance of glioma cells to treatment. The mechanism is based on activation of signaling pathways that promote cell survival, such as PI3K/Akt and MAPK/ERK, and stabilization of the factor HIF-1α, which promotes angiogenesis and hypoxia within the tumor. It has also been shown that NO can increase the expression of transcription factors such as NF-κB, which potentiates inflammation and stimulates the expression of genes associated with invasiveness and resistance to apoptosis. In contrast, at high concentrations, usually obtained experimentally or under intense oxidative stress, NO and the resulting peroxynitrite can act cytotoxic against cancer cells, leading to DNA damage, lipid peroxidation, and cell death. However, under real brain tumor conditions, the expression of inducible iNOS is usually moderate, which promotes the maintenance of an environment conducive to tumor progression. Numerous studies have shown that high iNOS expression in gliomas, particularly in GBM, correlates with poor prognosis and shorter patient survival. In addition, NO affects immune cells infiltrating gliomas—it promotes immunosuppression by, among other things, promoting the M2 phenotype in microglia and macrophages, as well as inducing T-regulatory cells to evade the anti-tumor response. Because of these properties, the iNOS/NO pathway represents a potential therapeutic target—research is underway on iNOS inhibitors and strategies to modulate NO production in the context of glioblastoma combination therapy [63,64,65].

### 3.2. NO and PDT

The most recent studies in the author’s laboratory have provided the first evidence that even low levels of NO, endogenously synthesized by cancer cells in response to photodynamic therapy (PDT)-induced stress, may play a key role in the development of resistance to phototoxicity induced by this treatment [66,67]. Experiments performed on the COH-BR1 cell model, a breast cancer cell line, consisted of sensitizing cells by induction of protoporphyrin IX (PpIX) using 5-aminolevulinic acid (ALA), which is a heme precursor and effectively increases the accumulation of the photosensitizer. Analysis of the subcellular localization of PpIX revealed its predominant accumulation in mitochondria, which were the main target of photodynamic damage during subsequent light exposure. Cells were irradiated with broad-spectrum visible light and moderate fluence (1–2 J/cm^2^), which corresponds to parameters often used in therapeutic conditions. After irradiation, a rapid and significant increase in the levels of mRNA transcript for iNOS and the iNOS protein itself was observed—reaching a 4–5-fold increase compared to the control group. Importantly, this induction was long-lasting, persisting for at least 20 h after termination of light exposure [67]. These results suggest that iNOS expression and associated NO production are not merely a consequence of cell damage, but constitute an active, adaptive response of the tumor to photodynamic stress. In this context, NO may act as a cytoprotective factor, protecting cells from apoptosis and supporting their survival and further development. This mechanism may significantly contribute to the ineffectiveness of PDT treatment in some clinical cases and indicates the need for further studies on the possibility of pharmacological modulation of the iNOS/NO pathway in the context of increasing the efficacy of photodynamic therapy.

**Figure 4:**
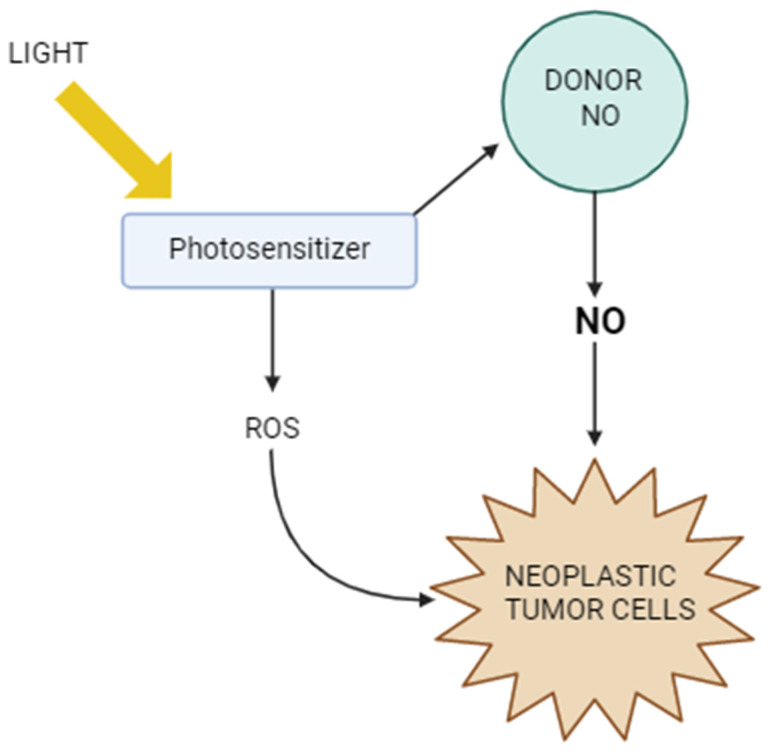
Photodynamic therapy vs. nitric oxide.

Figure 4 shows the mechanisms of action of PDT in the context of NO. It illustrates how light activates the photosensitizer, which generates reactive oxygen species, leading to cancer cell damage. At the same time, it is shown how nitric oxide can modulate the efficacy of PDT—both by supporting the therapeutic effect, e.g., by inducing oxidative stress and immunogenic cell death, and by tumor defense mechanisms, such as inhibiting lipid peroxidation or promoting cancer cell migration. The figure thus highlights the complex role of NO as a factor influencing the tumor response to photodynamic therapy.

High levels of NO can be used by cancer cells as an adaptation mechanism to environmental stress, including oxidative stress and hypoxia. This knowledge has led researchers to develop therapeutic strategies that deliberately exploit the presence of NO in tumors—both as a therapeutic target and as an activating agent for new forms of anticancer drugs. One of the most promising classes of compounds in this context are porphyrins—macrocyclic organic structures that exhibit high photoreactivity and have been used for years as photosensitizers in PDT. Their ability to generate singlet oxygen (^1^O_2_) upon activation with light is the basis for their cytotoxic effect on cancer cells. In addition, porphyrins exhibit fluorescent properties, which makes them valuable tools in molecular imaging. In order to exploit the selective activation of porphyrins by NO, Bandyopadhyay’s team designed an innovative series of compounds called NOxPorfins. These chemically modified porphyrins have been designed so that their photodynamic and fluorescent properties are “dormant” until they come into contact with pathological levels of NO in the tumor environment. The key element of this technology is the use of an o-phenylenediamine liberating group, which reacts with NO (or more precisely with its derivative, N_2_O_3_) in a cyclization reaction, leading to the formation of a fluorescent triazole [68].

The main representative of this series, NOxPorfin-1, showed an exceptionally strong response to the presence of NO—the fluorescence intensity increased 7.5-fold, and the efficiency of ^1^O_2_ generation increased as much as 70-fold after activation. In vitro studies confirmed the high selectivity of the reaction towards NO and not other reactive nitrogen or oxygen species. Further biological tests showed that NOxPorfin-1 exerts a strong cytotoxic effect under photodynamic conditions on breast cancer and non-small cell lung cancer cells. In vivo, the compound demonstrated the ability to effectively and locally destroy breast tumors in mice, without causing damage to healthy tissue. In a second model study, based on human lung cancer, NOxPorfin-1 completely stopped tumor growth and inhibited its further progression [68,69].

The ability of NOxPorphin-1 to precisely, locally activate in the presence of NO and its potent therapeutic properties make this compound—and the entire class of NO-reactive porphyrins—represent a new direction in precision oncology. These results are an important step towards the development of intelligent therapies that not only target the tumor but also use its own pathological molecular signals as triggers of the therapeutic effect. The potential use of NOxPorphins in the treatment of metastases—especially those characterized by high iNOS activity—opens new possibilities for second-generation photodynamic therapy, characterized by high specificity and limited side effects. Integrated therapeutic strategies combining classical PDT with targeted delivery of NO are becoming a subject of growing interest in the context of modern cancer treatment methods. Photodynamic therapy is based on the activation of photosensitizers using light of an appropriate wavelength, leading to the generation of reactive oxygen species (ROS), which cause structural and functional damage in cancer cells. Importantly, activation of photosensitizers can also lead to local production of NO, and the level of this signaling gas within the tumor and its microenvironment is crucial for the efficacy of PDT [70].

NO can modulate vascular permeability, induce an inflammatory response, and, at appropriate concentrations, sensitize cancer cells to stressors. In response to these relationships, Aikelam’s research team developed a new therapeutic system based on silicone phthalocyanine (SiPc)—a known second-generation photosensitizer—conjugated with a nitric oxide donor: 3-benzenesulfonyl-4-(1-hydroxyethyl)-1,2,5-oxadiazole-2-oxide (NO-donor). This combination was named SiPc-NO. In order to obtain a biocompatible form, this compound was formulated as self-assembled nanoparticles (SiPc-NO@NPs) by the solvent precipitation method. To ensure specificity towards cancer cells, the surface of the nanoparticles was modified with the RGD (arginine–glycine–aspartate) peptide, known for its high affinity for α_vβ_3 integrins, which are overexpressed on the surface of many types of cancer cells. The final product, designated as SiPc-NO@RGD NPs, was subjected to detailed characterization in terms of physicochemical stability, NO release efficiency in response to light, ROS generation, and in vitro phototoxicity. Studies conducted on two breast cancer cell lines—human (MCF-7) and mouse (4T1)—showed that after exposure to light, SiPc-NO@RGD NPs achieved high efficiency of ROS photogeneration and effectively induced cancer cell death [71]. The system also demonstrated fluorescent properties, enabling its use in biofluorescent imaging, making it a multifunctional tool, combining diagnostic (bioimaging), therapeutic (PDT), and immunomodulatory (NO release) properties [71,72]. In light of the obtained results, SiPc-NO@RGD NPs constitute an innovative therapeutic platform, offering the possibility of local, targeted, and controlled release of NO and photodynamic destruction of cancer cells. This approach may find application, especially in the treatment of tumors with difficult localization or resistant to classical therapies, and also constitutes a basis for the development of new, synergistic therapeutic methods integrating phototherapy, immunotherapy, and molecular imaging. PDT does not always lead to the complete elimination of cancer cells, which results, among others, from uneven light delivery and inhomogeneous tumor vascularization. As a consequence, some cells may survive the therapy and acquire features of a more aggressive phenotype, such as accelerated growth or increased migration capacity. This phenomenon was studied by the authors’ team on the model of human prostate cancer PC3 cells sensitized mitochondrially by ALA-induced PpIX [58,73]. It has been shown that after irradiation, there is a significant increase in iNOS expression (reaching up to eight times the initial value after 20 h), which correlates with increased resistance to apoptosis and increased cell proliferation, a phenomenon inhibited by the use of iNOS inhibitors (1400W) and an NO scavenger (cPTIO) [56]. There is now a wealth of evidence indicating that both constitutively and inductively expressed iNOS and its NO can reduce the efficacy of PDT against cancers and, in cases of incomplete tumor eradication, contribute to disease progression. It should be emphasized that both the uptake of the photosensitizer or its proforms, as well as the delivery of light and oxygen availability within the tumor, are usually uneven. As a result, individual cells in the tumor are exposed to PDT to varying degrees—some undergo severe oxidative stress, while others may remain relatively unaffected, functioning as so-called “passive observers” [74]. In vitro studies by Girotti et al. have shown that cancer cells that survived PDT exhibit significantly increased iNOS expression, which leads to continuous NO production [50]. Nitric oxide promotes the survival of these cells and their proliferation. Importantly, NO also diffuses into neighboring, undamaged cells, inducing iNOS expression in them and thus initiating a cascade of growth and increased migration potential. Such action may contribute to the development of a more aggressive cancer phenotype and promote the ability to metastasize, which is a particularly undesirable effect of therapy. The described phenomenon resembles a relay mechanism, in which NO production by one cell population induces a similar response in the next ones, leading to a wide spread of the signal within the tumor. This mechanism is analogous to the well-known “bystander” effect described earlier in the context of cell exposure to ionizing radiation. It has also been shown that this phenomenon can affect not only bystander cells but also the internal target population, which was confirmed in experiments with separate cell fractions [75].

### 3.3. Role of iNOS Inhibitors in Cancer Therapy

Inducible iNOS is an enzyme encoded by the NOS2 gene, whose expression is usually low in healthy tissues, but can be strongly induced under the influence of pro-inflammatory factors such as cytokines (TNF-α, IFN-γ), lipopolysaccharides, and oxidative stress. In many malignancies, overexpression of iNOS is observed, resulting in the production of large amounts of nitric oxide (NO). For example, in breast cancer, immunohistochemical studies have shown a strong correlation between high levels of iNOS and aggressive tumor phenotype and poor prognosis of patients. Analogous observations have been reported in lung cancer, where iNOS overexpression is associated with increased angiogenesis and greater metastatic capacity of cells [76,77].

The mechanism of iNOS overexpression in cancer is complex and involves activation of the NF-κB and STAT3 signaling pathways, which are often overactive in cancer cells and promote the expression of pro-inflammatory and proliferative genes. Consequently, NO produced by iNOS acts as a mediator that promotes tumor progression. NO is a molecule with unique properties whose role depends on its local concentration and time of action. At low concentrations, NO has an antiproliferative effect and can induce apoptosis of cancer cells by activating caspases and damaging mitochondrial DNA. However, under conditions of chronic inflammation and high iNOS expression, NO reaches concentrations that promote cancer cell survival and growth. NO at high concentrations can react with oxygen radicals to form reactive nitrogen species (RNS), which cause DNA mutations, epigenetic changes, and inhibit DNA repair mechanisms, contributing to genetic instability of the tumor [78,79].

In addition, NO modulates the activity of transcription factors such as NF-κB and HIF-1α. HIF-1α is crucial in the response to hypoxia in the tumor and stimulates angiogenesis through induction of VEGF expression. In addition, NO inhibits the function of anti-tumor T cells and macrophages, leading to immunosuppression and enabling tumor cells to evade immune control [65].

Due to the multifaceted role of iNOS in cancer progression, its selective inhibition has become the focus of intense pharmacological research. Inhibitors such as 1400W and L-NIL show high selectivity toward iNOS and inhibit NO production without significantly affecting other isoforms of nitric oxide synthase (eNOS and nNOS), minimizing potential side effects. In studies on colon cancer cell lines, 1400W treatment resulted in inhibition of proliferation and induction of apoptosis of tumor cells. In animal models of mice with implanted lung cancer tumors, administration of iNOS inhibitors reduced the formation of new blood vessels and reduced tumor size. Importantly, the use of iNOS inhibitors showed synergistic effects in combination with chemotherapy and radiation therapy, increasing their efficacy and improving animal survival [80,81].

Despite promising results from preclinical studies, the introduction of iNOS inhibitors into routine anticancer therapy faces significant obstacles. A key challenge is the biphasic effect of NO—complete inhibition of NO production can disrupt physiological processes such as blood pressure regulation and immune function. In addition, the diversity of cancer types and the heterogeneity of iNOS expression at different stages of the disease require a personalized approach [82,83].

Accordingly, researchers are proposing novel therapeutic approaches, including nanoparticle carriers that enable targeted delivery of iNOS inhibitors directly to the tumor microenvironment. Preliminary results from these studies indicate a reduction in side effects and increased efficacy of therapy. At the same time, studies are underway to identify predictive biomarkers to select patients who will benefit most from iNOS inhibitor therapy [84,85,86].

Moreover, NO secreted by surviving cells can stimulate the migration and invasion of both these cells and bystanders—even in distant tumor regions.

## 4. Discussion

Studies have shown that PDT stimulates iNOS/NO overexpression in surviving cancer cells, which promotes their resistance and increased migration and invasion [87,88,89,90]. Girotti et al. describe a mechanism in which iNOS is induced after PDT and produces NO, which inhibits lipid peroxidation in cell membranes—the primary cytotoxic process of PDT [91,92].

Moreover, NO secreted by surviving cells can stimulate the migration and invasion of both these cells and bystanders—even in distant tumor regions [87,92]. In glioblastoma models, U87 and U251 showed that NO activates NF-κB, PI3K/Akt pathways and even proteins such as Survivin and Brd4, contributing to growth and aggressiveness [88,89,93,94].

Literature reviews indicate that adding selective inhibitors of inducible iNOS, such as 1400W, cPTIO, or L-NAME, to PDT significantly increases the efficacy of the treatment by enhancing cytotoxicity and limiting the migration of cancer cells. The 1400W inhibitor shows high selectivity for iNOS, without affecting other NO synthase isoforms, which minimizes the risk of adverse effects. Alternatively, cPTIO acts as a scavenger of free NO molecules, reducing its pro-migration and pro-invasion effect on bystander cells. In addition to direct inhibition of enzymatic activity, modulation of the NO pathway can also be achieved at the level of NOS2 gene expression. In this context, promising results are brought by BET protein inhibitors, such as JQ1, which block the recruitment of transcription factors to the NOS2 promoter, resulting in a decrease in iNOS levels and NO production. Combination therapies combining PDT with pharmacological modulation of the NO pathway may increase the effectiveness of cancer treatment by simultaneously enhancing the cytotoxic effect and limiting resistance mechanisms, which makes this strategy attractive for further preclinical and clinical studies [93,95].

The latest research on PDT focuses on the development of innovative photoadditive compounds called NO-releasing photosensitizers (NO-PSs), which, when activated by light, are capable of precise, controlled release of NO directly in the tumor microenvironment. This precise spatial and temporal control of NO release allows us to overcome the key limitations of traditional PDT, especially related to oxygen deficiency (hypoxia), characteristic of most tumors. NO plays a double, synergistic role in this process—on the one hand, it intensifies the cytotoxic effects generated by PDT, increasing nitroxidative stress in tumor cells; on the other hand, it counteracts hypoxia by improving perfusion and microcirculation within the tumor. Improved blood supply and oxygenation of the tumor increases the availability of oxygen necessary for the generation of reactive oxygen species (ROS) by photosensitizers, which enhances their therapeutic efficacy [96].

In addition, NO, as an important immune mediator, stimulates the activation of various elements of the immune system, including T lymphocytes, macrophages, and dendritic cells, which enhances the immunogenic effect of PDT and promotes the induction of a long-lasting anti-tumor response. As a result, NO-PSs not only cause direct tumor cell death but also support the recruitment and activation of immune cells, which is crucial for tumor control and elimination. Studies conducted on both in vitro and in vivo models have shown that NO-PSs are effective even under hypoxic conditions, which usually limit the effectiveness of traditional PDT and other cancer therapies. Cancer cells in a hypoxic microenvironment often show increased resistance to treatment, and NO released locally by NO-PSs can overcome this resistance, which opens new therapeutic perspectives. This approach is particularly promising in the treatment of tumors with poor blood supply and high hypoxia, which are difficult to combat with standard methods [97,98]. An interesting example is the unified ZnPc-2NO and ZnPc-4NO particles, which, after entering cancer cells, react with intracellular glutathione, which leads to the release of NO. This results in a decrease in oxygen consumption by mitochondria, a decrease in HIF-1α activity and increased oxygen availability for the generation of ROS during PDT, which results in the intensification of oxidative stress and tumor death, also under hypoxic conditions [99].

The study by Xu et al. presented an innovative M1@PAP system based on M1-type macrophage membrane vesicles loaded with a type I photosensitizer and siRNA against PD-L1 (siRNA-PD-L1) [99]. The photosensitizer, a conjugate of pyropheophorbide (PPA) and multiple polyl-arginine (Arg_9_), plays a triple role here: first, it acts as a light sensor activating the photogeneration of superoxides even under hypoxic conditions—characteristic of hypoxic tumors; second, Arg_9_ acts as an NO donor, which leads to the inhibition of cancer fibroblast (CAF) activity and loosening of the dense extracellular matrix, significantly improving the penetration of nanoparticles; third, the released NO and M1 macrophage factors reprogram macrophages within the tumor from the M2 to M1 phenotype, which promotes immunomodulation and enhances the response to PD-L1 therapy [100]. The M1@PAP system enables effective combined photodynamic and immunological therapy, showing promising anti-tumor activity in hypoxic conditions. By breaking down physical (hyperdense ECM) and immunological (immunosuppressive microenvironment) barriers, M1@PAP acts as a multifunctional therapeutic nanoplatform that synergistically improves penetration, cytotoxicity, and activation of the immune system.

In recent years, the development of NIR-activated nanoplatforms such as P1-CapNO NPs has brought about a breakthrough in photothermal and NO therapy, especially in hypoxic conditions. A 2023 study published in the *Journal of Nanobiotechnology* describes P1-CapNO NPs—polymeric nanoparticles equipped with a thermosensitive NO component and a photosensitizer with a maximum absorption at ~790 nm (molar absorption 2.4 × 10^5^ M^−1^ cm^−1^), and a photothermal conversion exceeding 23.8%. Under the action of an 808 nm laser, they release NO at a concentration of about 1.3 µM, regardless of the amount of oxygen in the environment. In in vitro and animal hypoxia models, the combination of photothermal effect and cytotoxic NO action led to a significant reduction of tumors, confirming the effective breakthrough of hypoxia barriers [101].

In parallel, work on porphyrin-MOF systems with nicorandil showed that the reaction of nicorandil with glutathione inside the tumor leads to the controlled release of NO, reducing hypoxia and depleting GSH. This effect, combined with the generation of ROS, promotes the production of peroxynitrite (ONOO^−^), which strongly damages breast cancer cells [102]. In addition, extensive reviews of NO-assisted photothermal therapy technologies demonstrate the efficacy of various platforms (PEG-PAu@SiO_2_-SNO, CuS-PEI-TPP, etc.), operating in the 808–1064 nm range, which enhance cancer cell killing through the synergistic action of thermal and NO [103].

## 5. Conclusions

NO is a gaseous transmitter with complex and multifaceted effects, whose biological activity includes both protective and cytotoxic effects depending on its local concentration, source of synthesis, and oxidative environment. Understanding these diverse mechanisms is crucial for the development of targeted therapeutic interventions in diseases associated with dysfunction of NO signaling pathways. The action of nitric oxide in photodynamic therapy is a phenomenon that has only recently gained greater attention from researchers, but still requires further exploration, especially in the field of molecular mechanisms that underlie its influence on the effectiveness of PDT. The final understanding of the role of NO in the context of this therapy could contribute to the development of new therapeutic strategies that would improve the effectiveness of cancer treatment. PDT involves the use of photosensitizers (PSs), which are activated by light of an appropriate wavelength. Most of these sensitizers are amphiphilic, which means that they have both a hydrophobic and a hydrophilic part, which allows them to be embedded in the structures of cell membranes. Due to this property, photosensitizers accumulate mainly in cancer cells, in particular, in their cell membranes. When these sensitizers are activated by light, they trigger photochemical reactions that lead to the generation of reactive oxygen species, such as oxygen radicals and superoxides. These reactive molecules can then lead to structural and functional damage of cancer cells. However, the cell membranes in which photosensitizers are concentrated also contain unsaturated lipids, which are particularly sensitive to peroxidation after photoactivation. Lipid peroxidation leads to the formation of damage that can affect the integrity of the cell membrane and make the cell more susceptible to further damage or metabolic disorders. On the other hand, lipid peroxidation can also lead to the generation of new signaling molecules, such as nitric oxide, which in this case can have both protective and potentially harmful effects, depending on the context. Surviving cells can produce more NO, which can affect nearby tumor or endothelial cells by stimulating their proliferation, migration, or invasion. Therefore, a better understanding of this mechanism, including the ways in which NO influences the therapeutic effect of PDT, may lead to the development of more precise and targeted treatment strategies that address this additional signaling pathway.

## Figures and Tables

**Table 1:** Biological significance of nitric oxide [36,37,38].

Concentration	Description	Biological Function
~100 nM	Very low; typical resting levels in many cell types	Basic vasodilation, immune surveillance, basic neurotransmission
300–500 nM	Slight increase during cell signaling	Endothelial NO production (eNOS)—regulation of vascular tone, weak immune activation
>1 µM	High concentrations	Associated with inflammation, immune system activation, potential cytotoxicity

**Table 2:** The role of nitric oxide (NO) in a particular type of cancer [39,40,42,43,44,45,46,47,48,49].

Cancer Type	Level No (INOS)	Biological Effect	Related Glasses/molecules
BREAST CANCER	Low–medium (nanomolar)	Promotion of growth and migration	EGFR, PI3K/Akt, ERK1/2, S-nitrosation of PTEN, Bcl-2
PROSTATE CANCER	Low–Medium	Supporting tumor survival and progression	S-nitrosation of Bcl-2, inactivation of PTEN
BLADDER CANCER	Low–Medium	Activation of pro-growth pathways	HIF-1α, sGC, PI3K/Akt
CERVICAL CANCER	Low–Medium	Increased angiogenesis and cell survival	HIF-1α, S-nitrosation of JNK/ASK1
GLEJAK (BRAIN)	Low–Medium	Growth, migration, resistance	EGFR, PI3K/Akt, ERK1/2, S-nitrosation
CANCERS WITH P53 MUTATED	High (micromolar)	Increased aggressiveness and migration	Overexpression of iNOS, lack of growth inhibition by p53
MACROPHAGES (INOS) IN TME	High	Cytotoxicity against cancer cells	NO as an effector of the immune response (micromolar level)

## Data Availability

No new data were created or analyzed in this study.
